# Ambivalent identification mediates the relationship between organizational justice and stress

**DOI:** 10.3389/fpsyg.2023.1260768

**Published:** 2023-11-20

**Authors:** Valeria Ciampa

**Affiliations:** Department of Psychology, Sapienza University of Rome, Rome, Italy

**Keywords:** ambivalent identification, organizational justice, stress, social identity, work-related burnout, client-related burnout, interpersonal strain, physical symptoms

## Abstract

The present study aims to examine the relationship between organizational justice and employee stress through the lenses of social identity theory and the ambivalent identification process. The research hypotheses assume that employees working in organizational environments with low levels of justice could experience more stress, and this relationship is also mediated by ambivalent identification. In other words, the mediating mechanism of this relation posited that low levels of organizational justice were associated with high levels of ambivalent identification, which in turn increased levels of work-related stress. Across a field study in several organizations from healthcare sectors, results confirmed that employees treated with less fairness experienced high ambivalence toward their organization, which increased their perception of stress, i.e., work-related burnout, client-related burnout, physical symptoms, and interpersonal strain at work. Furthermore, results supported only a full mediation model, in which the direct relationship between organizational justice and stress was not significant. The present results make an important contribution to the research literature on justice: the inclusion of the mediator variable, namely, ambivalent identification, drops the expected direct effect of organizational justice on stress, suggesting a call for action in adopting the social identity perspective in addition to organizational justice models, and specifically introducing the study of a detrimental form of identification, such as ambivalent identification. Limitations and practical implications of the study were discussed.

## 1 Introduction

What could be more frustrating than a colleague receiving credit for a job you did? It is generally accepted that when facing unfair situations, the immediate reaction could be anger or negative emotions (Fox et al., 2001; Pérez-Rodríguez et al., 2019), but sometimes such conditions could have even worse effects on both individuals and organizations, like employee turnover intention or withdrawal (Conlon et al., 2005; Moon, 2017; Zhou et al., 2022). Nevertheless, if employees have a strong desire to keep working with the perpetrator of the injustice, or with the group of colleagues they admire, their reactions or consequences could not be so consistent or linear. In literature, the investigation of employees’ reactions more rarely takes into account the complexity of contradictory inner feelings that arise from a disjunction between people’s aspirations and desires and the actual possibility of realizing them. On the other hand, studies over the past three decades have provided important information about the negative effects of organizational (in)justice or unfairness (e.g., Colquitt et al., 2001), across cultures and countries, specifically on health and psychological well-being (Fischer, 2013; Silva and Caetano, 2016). Most of the literature recognizes that a perceived lack of organizational justice or fairness represents an important psychosocial risk factor particularly related to high levels of stress, that may even lead to coronary heart disease (Sara et al., 2018), inducing a profound negative impact on employee well-being. Hence, understanding the relationship between organizational justice and stress is vital for organizations aiming to enhance employee well-being and organizational performance. By identifying how organizational injustice can increase employee stress levels, organizations can develop targeted interventions and policies to promote a fair and supportive work environment, limiting the negative consequences for the whole organization. Accordingly, the purpose of this investigation is not only to explore the relationship between organizational justice and stress, but also to propose an explanatory mechanism for this link. Thus, the present study has two aims. First, testing the direct relationship between organizational justice and different outcomes of stress. Second, exploring how this relationship works, hypothesizing the mediating role of ambivalent identification between the justice-stress relationship, that is adopting the social identity perspective to empirically evaluate whether the mediator explains why the effect of organizational justice on stress happens.

To date, the field of organizational justice has produced different models of studying employee perceptions of fairness and their correlates (see, e.g., Greenberg, 1987; Greenberg and Colquitt, 2013; Cropanzano and Ambrose, 2015). Of particular concern for employee health is the relationship between organizational justice and work-related stress, which has been widely supported by empirical literature (see Cropanzano and Wright, 2011). For example, researchers found that low perceived justice was associated with sickness absence from work, in presence of poor self-rated health, and minor psychiatric disorders (Elovainio et al., 2002). Fox et al. (2001) used a theoretical job stress framework considering organizational justice as a fundamental job stressor that could lead to behavioral strain responses, such as counterproductive work behaviors, and negative emotions (Fox et al., 2001). The link between organizational justice and perceived stress was also found in a study by Judge and Colquitt (2004), who examined the mediational role of work–family conflict in this relationship. Another study on this link confirmed that two specific dimensions of organizational justice, namely, distributive and procedural, were related to a general measure of job stress (Lambert et al., 2007). The organizational justice models have also been applied to examine their association with stress-related disorders, like sickness absence (Head et al., 2007), but also burnout, and depression (Liljegren and Ekberg, 2009; Ndjaboulé et al., 2012; Eib et al., 2018). Overall, these studies provide evidence for the negative impact of organizational (in)justice on employee health and well-being, although much of the literature mainly focuses on mental health and psychiatric disorders (e.g., Elovainio et al., 2009; Ndjaboulé et al., 2012; Fischer et al., 2014). As a matter of fact, only a limited number of studies have examined the association between organizational justice and specific measures of work-related stress. Accordingly, based on the literature reviewed here, and the consideration that more attention should be paid to unexplored outcomes (Cachón-Alonso and Elovainio, 2022), the following hypothesis was posited:

*Hypothesis 1*: Organizational justice will be negatively related to perceptions of stress, i.e., work-related burnout, client-related burnout, physical symptoms, and interpersonal strain at work.

With regard to the mechanisms that can explain the association between organizational justice and stress, there is still very little scientific understanding of this aspect. Although some research has explored this link using different theoretical frameworks (see, e.g., Pérez-Rodríguez et al., 2019; Murtaza et al., 2023), much of the perspective adopted considers employees as isolated agents within the organization in which they work, neglecting that they are immersed in social contexts. As Tajfel and Turner stated: “In our judgments of other people, […] in our work relations, in our concern with justice, we do not act as isolated individuals but as social beings who derive an important part of our identity from the human groups and social categories we belong to; and we act in accordance with this awareness” (Tajfel et al., 1984, p. 5). Building on social identity and self-categorization perspectives, we can thus explain why individuals interpret external stimuli as stressors, and what happens when they cannot rely on their internal resources – such as their strong identification with their organization – to cope with stress. According to this perspective, the organizational identity becomes part of individuals’ self-concept (Tajfel and Turner, 1979; Turner et al., 1987), experiencing a particular type of social identity, that is, employee identification towards the organization (Ashforth and Mael, 1989), protecting individuals from developing stress responses (Steffens et al., 2017; Ciampa et al., 2019). Therefore, considering that the definition of ourselves varies as a function of contexts, employees’ social identity cannot become salient when injustice and inequity characterize the organization and people are treated unfairly. In such conditions, indeed, the sense of “we” or “us” may not raise stronger than the sense of “I” or “me,” undermining the process to define “myself” as a group member (in contrast to outgroup members). Consequently, when the contextual conditions are not favorable, i.e., in the presence of organizational injustice, individuals could develop conflicting emotions and cognitions toward the organization, simultaneously identifying with some aspects while rejecting other aspects of their organization that they do not want to integrate into their self-definition, developing contradictory and ambivalent attachments (Pratt, 2000; Kreiner and Ashforth, 2004). Ambivalent identities may be elicited in such situations because individuals must struggle between a context discouraging their social identity (or rather not fostering it), and their implicit desire for belongingness. The social need of belonging to the ingroup is indeed a primary inner motivator that comes from the need to enhance social self-esteem and to achieve collective self-actualization, which is not less important than the need for personal self-actualization and self-esteem (Leavitt, 1995; Haslam, 2004, p. 386). Moreover, Ashforth et al. (2014) argue that organizational dualities are particularly likely to provoke ambivalence because they simultaneously promote opposite norms, values, and beliefs about what is and what is not acceptable within the ingroup (e.g., competition versus cooperation). For example, when the management establishes a collective goal, but employees are not treated fairly receiving different honors and payoffs based on the boss’ preferences, this could implicitly spread the idea that cooperation for a common goal does not benefit the whole group. Consequently, a violation of the explicit norms settled by the management about, e.g., rewarding a completed task, can induce employees to compete against each other, instead of cooperating for a common goal. Such dualities are thus a potential way to develop and spread “paradoxes of belonging” (Smith and Berg, 1987): all those situations that we can call injustices or unfair conditions can easily undermine the sense of “us” of the group, inducing ambivalent feelings, particularly in employees who do not benefit from unequal treatments.

About the consequences of ambivalent identification on employee health, this relationship has not been thoroughly examined in previous research within organizational psychology, despite the literature offering some general models of potential adverse effects of ambivalence (Pratt, 2000; Rothman et al., 2017; Zhao and Zhou, 2021; Wu et al., 2023). For example, a study by Ciampa et al. (2019) revealed that ambivalent identification was positively associated with ego depletion and emotional exhaustion (Ciampa et al., 2019). More specifically, their results showed that the negative association between positive organizational identification and strain was significant and stronger for employees experiencing low levels of ambivalence, meaning that the presence of ambivalence decreases the likelihood that a clear identification may protect against stress reactions (Ciampa et al., 2019). In the same vein, another recent study linked ambivalent leadership to different mental health outcomes in employees, i.e., depression, anxiety, vital exhaustion, and fatigue, both at within-group and between-group levels (Herr et al., 2022). In summary, according to the literature reviewed here, there are reasons to expect that ambivalent identification would mediate the relationship between (in)justice and stress, representing a detrimental factor for employee health. Therefore, the following two predictions were advanced:

*Hypothesis 2*: Organizational justice will be negatively related to ambivalent identification.

*Hypothesis 3*: The negative relationships among organizational justice and work-related burnout, client-related burnout, physical symptoms, and interpersonal strain at work will be partially mediated by ambivalent identification, which, in turn, will be positively associated with such stress outcomes.

Overall, the present study can contribute to the literature by highlighting the role of ambivalent identification, a specific form of identification – related to the simultaneous coexistence of opposite orientations toward the organization – still overlooked in this area of research. Moreover, the focus on this negative dimension can be crucial in designing preventive programs aimed to develop individual and organizational resources to counteract organizational injustice and its consequences.

## 2 Method

### 2.1 Participants and procedures

Several organizations from public and private healthcare sectors were involved in the present study. Specifically, the target sample consisted of employees working in public hospitals and private clinics accredited by the National Health System, where the typical organizational structure would usually be a combination of a hierarchical and departmental structure. These organizational characteristics allowed the possibility of studying organizational justice in the presence of a chain of command, where some levels are subordinate to another level, but employees are organized in wards that have their own tasks. Furthermore, to select similar environmental conditions, only wards with staff working with hospitalized patients were included, while emergency and intensive care units were excluded. The employees invited to participate in the study were physicians, medical technicians, nurses, and administrative staff, excluding the top level of administrative services, such as the board of directors, executive officers, presidents, and vice presidents. A letter of invitation to participate in the study was sent to managers, explaining the purpose of this investigation, and requesting that employees with the required characteristics be invited to complete an anonymous questionnaire containing the research measures. Participation was completely voluntary, and the questionnaire was delivered to the managers via a unique online link that included the informed consent materials, which explained the anonymous nature of the data collection and their rights as research participants, while not asking for any personal information. After accepting the informed consent, a total of 195 useful questionnaires were returned from eleven regions of Italy. The average age of respondents was 47.95 (*SD* = 10.14), and the vast majority were male: only 29% were female, 16% were missing. Over half of the participants were doctors (55%), medical technicians (16%), nurses (10%), and administrative staff (4%; 16% were missing). Finally, average organizational tenure was 14.8 years (*SD* = 9.95), and work experience was 19.4 years (*SD* = 10.97).

### 2.2 Measures

*Organizational Justice Index* was measured using the Italian version (Capone and Petrillo, 2016) of the 10-item scale from Hoy and Tarter (2004). A sample item was “*In this organization, all workers are treated fairly*.” Respondents indicated their agreement with each statement from 1 (*strongly disagree*) to 5 (*strongly agree*). The reliability of the scale was *α* = 0.92.

*Ambivalent Identification* was measured with the 6-item scale developed by Kreiner and Ashforth (2004), using the Italian version translated by Ciampa (2018) and Ciampa et al. (2019, 2021). A sample item was “*I have mixed feelings about my affiliation with this organization*.” Respondents indicated their agreement with each statement from 1 (*strongly disagree*) to 5 (*strongly agree*). The reliability of the scale was *α* = 0.83.

*Work-related Burnout* (CBI) was measured using the Italian version of the 7-item subscale from the Copenhagen Burnout Inventory (CBI; Kristensen et al., 2005; Avanzi et al., 2013). Respondents indicated how often they experienced each statement on a Likert scale from 1 (*never*) to 5 (*always*). A sample item was “*Are you exhausted in the morning at the thought of another day at work*.” The reliability of the scale was *α* = 0.82.

*Client-related Burnout* (CBI) was measured using the Italian version of the 6-item subscale also selected from the Copenhagen Burnout Inventory (CBI; Kristensen et al., 2005; Avanzi et al., 2013). Participants indicated how much they experienced each statement on a Likert scale from 1 (*not at all*) to 5 (*extremely*), according to the original scale. A sample item was “*Do you find it frustrating to work with clients?*”. The reliability of the scale was *α* = 0.85.

*Physical Symptoms Inventory* (PSI) was measured with a 13-item scale developed by Spector and Jex (1998). Items included symptoms such as “*Headache*,” “*Backache*,” “*Trouble sleeping*,” and so on. Participants indicated how often they experienced each symptom from 1 (*not at all*) to 5 (*every day*). The reliability of the scale was *α* = 0.84.

*Interpersonal Strain at Work Scale* (ISW) was measured with the Italian 6-item scale (Borgogni et al., 2012), rated on a 7-point frequency scale ranging from 0 (*never*) to 6 (*daily*). A sample item was “*At work, I treat others in a cold and detached manner*.” The reliability of the scale was *α* = 0.88.

*Control* var*iables.* Based on previous literature, several sociodemographic variables, namely, age, sex, work experience, organizational tenure, and working hours, were included in the questionnaire as control variables that may potentially influence stress outcomes and employee well-being (see, e.g., Purvanova and Muros, 2010; Boyas et al., 2013; Antao et al., 2022; Lee et al., 2022; Schroeder et al., 2022). We used one-item measures for all control variables. Working hours were measured with the question “*How many hours do you work in total during a week?*,” and employees were asked to choose an option among “20–29,” “30–39,” “40–49,” “50–59,” “60–69” hours. However, preliminary data analysis showed that none of the control variables was significantly associated with the dependent variables, apart from working hours, which showed a statistically significant difference only between 30–39 h and 50–59 h on work-related burnout (*F*_(4,160)_ = 3.830, *p* < 0.01, *η*^2^ = 0.09), but not on other dependent variables. Accordingly, the hypothesized model was performed by controlling for working hours, and then it was compared to the same model without this control variable. From the comparison it emerged that standardized coefficients of the independent variables with and without the control variable differed by less than 0.1, therefore differences were considered negligible (Becker, 2005), as suggested by Becker et al. (2016) recommendations. Accordingly, only the results without the control variable were reported.

### 2.3 Analytic strategy

Structural equation models with latent variables were carried out with Mplus 8 (Muthén and Muthén, 1998-2017). Due to a moderate violation of the normality of some variables, the Weighted Least Squares Mean and Variance (WLSMV) estimation was used (Asparouhov and Muthén, 2010), which does not assume normally distributed variables and provides the best option for modeling ordered data (Beauducel and Herzberg, 2006; Brown, 2006). First, the hypothesized model was performed and compared with alternative models by evaluating goodness-of-fit indices (Kline, 2011). Second, to determine the size and significance of the indirect effects, estimates were bootstrapped 10,000 times from the final structural model and analyzed their standardized estimates along with the corresponding 95% confidence intervals (MacKinnon, 2008).

## 3 Results

### 3.1 Test of measurement model

To test the measurement model, a confirmatory factor analysis was performed using WLSMV estimation, consisting of the hypothesized six latent variables (i.e., organizational justice index, ambivalent identification, work-related burnout, client-related burnout, physical symptoms, and interpersonal strain at work) and their respective item-level indicators. The fit of this model was then compared with three plausible alternative models: one that combined the four outcomes of stress into a single factor, one that combined the independent variable and the mediator into a single factor, and finally, one that combined all the variables into a single factor. Based on the model fit indices shown in Table 1 and the robust Chi-Square Difference Testing of the nested models (Asparouhov and Muthén, 2006), the best-fitting model appeared to be the hypothesized six-factor model, since the models with constrained parameters appeared statistically different from the hypothesized model, indicating that the model with less parameters should be preferable. Moreover, each indicator had statistically significant factor loadings (*p* < 0.001) on its assigned dimension, confirming that a model considering six latent variables was appropriate.

**Table 1:** Confirmatory factor analysis results for the test of the measurement model.

Model	*χ*^2^	df	CFI	TLI	RMSEA (90% C.I.)	SRMR	Chi-square test for difference testing	df	*p*-Value
Hypothesized 6-factor model	1387.152*	1,065	0.966	0.964	0.039 (0.033–0.045)	0.079	–	–	–
Model combining OJI and AID (5 factors)	1853.632*	1,070	0.917	0.913	0.061 (0.057–0.066)	0.101	153.464	5	0.000
Model combining WRB, CRB, PS, IS (3 factors)	2249.037*	1,077	0.876	0.870	0.075 (0.071–0.079)	0.119	263.627	12	0.000
Model combining OJI, AID, WRB, CRB, PS, IS (1 factor)	6123.326*	1,080	0.467	0.443	0.155 (0.151–0.159)	0.244	1059.986	15	0.000

OJI, Organizational Justice Index; AID, Ambivalent Identification; WRB, Work-Related Burnout; CRB, Client-Related Burnout; PS, Physical Symptoms; IS, Interpersonal Strain at work. The chi-square statistic reflects the difference test between the hypothesized tested model and the respective nested models; *p < 0.001.

### 3.2 Descriptive statistics and hypothesis tests

Table 2 presents the descriptive statistics, scale reliabilities, and intercorrelations among the study variables.

**Table 2:** Descriptive statistics reliabilities (Cronbach’s alpha) and intercorrelations of all study variables.

	M	SD	Sk	Ku	*α*	1	2	3	4	5
1. Organizational justice index	3.70	1.03	−0.16	−0.82	0.92	–				
2. Ambivalent identification	2.61	0.73	−0.06	−0.39	0.83	−0.39**	–			
3. Work-related burnout	2.57	0.71	0.06	−0.46	0.82	−0.19**	0.33**	–		
4. Client-related burnout	2.26	0.80	0.28	−0.87	0.85	−0.14	0.36**	0.56**	–	
5. Physical symptoms inventory	1.72	0.57	1.37	2.58	0.84	−0.12	0.24**	0.43**	0.36**	–
6. Interpersonal strain at work	1.93	0.79	1.91	5.88	0.88	−0.05	0.27**	0.28**	0.39**	0.18*

M, mean; SD, standard deviation; Sk, skewness; Ku, kurtosis; *α*, Cronbach’s alpha; *p < 0.05, **p < 0.01.

To test the hypothesized model, a structural equation model (Model 1) was performed using the WLSMV method of estimation. In this model, the direct and indirect effects were posited among organizational justice and the four outcomes. This model showed an adequate fit to the data: *χ*^2^_(1065)_ = 1387.152, *p* = 0.000; CFI = 0.966; TLI = 0.964, RMSEA = 0.039, C.I. = 0.033–0.045; SRMR = 0.079. However, contrary to Hypothesis 1, there were not statistically significant effects between organizational justice and work-related burnout (*β* = −0.07, *p* = 0.351), as well as client-related burnout (*β* = −0.04, *p* = 0.652), physical symptoms (*β* = −0.01, *p* = 0.890), and interpersonal strain (*β* = −0.10, *p* = 0.146). Nevertheless, as predicted by Hypothesis 2, organizational justice exerted a negative significant effect of −0.42 (*p* < 0.001) on ambivalent identification.

Considering the non-significant effects of organizational justice on each outcome, an alternative structural equation model (Model 2) was performed, in which the direct effects of organizational justice on work-related burnout, client-related burnout, physical symptoms, and interpersonal strain were constrained to be zero, or rather, the effect of organizational justice on all outcomes would be fully mediated by ambivalent identification. This model showed an excellent fit to the data: *χ*^2^_(1069)_ = 1361.166, *p* = 0.000; CFI = 0.969; TLI = 0.967, RMSEA = 0.038, C.I. = 0.031–0.043; SRMR = 0.080. Furthermore, results from the chi-square test for difference testing between Model 1 and Model 2 were not significant (Δ*χ*^2^_(4)_ = 2.922; *p* = 0.571). Therefore, since parsimony is desirable in structural equation modeling (Preacher, 2006), Model 2 was preferred because the two models showed an equal level of fit to the data. Results of indirect effects are reported in Table 3.

**Table 3:** Results of the indirect effects.

Total and specific indirect effects	*β*	*SE*	95% C.I.
OJI → AID → Work-related burnout	−0.174*	0.048	[−0.278; −0.088]
OJI → AID → Client-related burnout	−0.161*	0.046	[−0.266; −0.073]
OJI → AID → Physical Symptoms Inventory	−0.128*	0.036	[−0.214; −0.068]
OJI → AID → Interpersonal Strain at work	−0.132*	0.038	[−0.214; −0.058]

Total, total effects, and specific indirect effects are equal since it is a full mediation model. OJI, Organizational Justice Index, AID, Ambivalent Identification. SE, standard error. 95% confidence intervals (95% CIs) are based on a *N* = 10,000 sample bootstrapping method; *p < 0.001.

**Figure 1:**
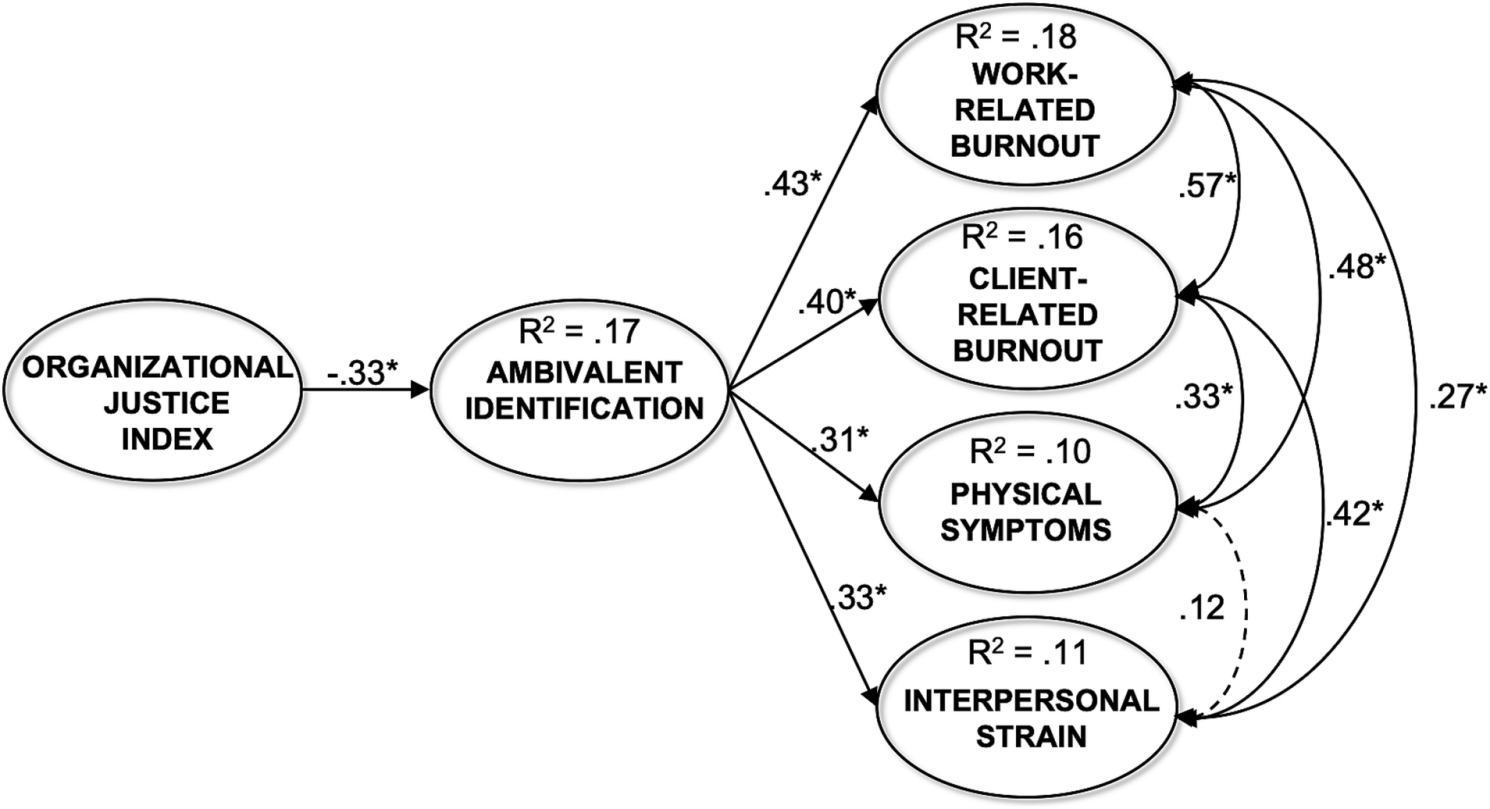
Results from the final structural equation model (Model 2). Coefficients are reported in a standardized form; *p < 0.001. The dotted line is a non-significant effect.

As can be seen in Figure 1, organizational justice exerted a negative significant effect of −0.41 (*p* < 0.001) on ambivalent identification, supporting Hypothesis 1. Ambivalent identification, in turn, exerted a positive significant effect on work-related burnout (*β* = 0.43, *p* < 0.001), client-related burnout (*β* = 0.40, *p* < 0.001), physical symptoms (*β* = 0.31, *p* < 0.001), and interpersonal strain (*β* = 0.33, *p* < 0.001), that is higher levels of ambivalent identification were associated to higher levels of stress on a moderate extent. Overall, the model explained the 17% of ambivalent identification variance and the 18% of work-related burnout variance, the 16% of client-related burnout variance, the 10% of physical symptoms variance, and the 11% of interpersonal strain variance. Results of the indirect specific effects of organizational justice to stress outcomes through the mediator are presented in Table 3.

## 4 Discussion

The present findings show that employees working in unfair organizational contexts can develop ambivalent identification toward their organizations, which, in turn, can lead to stress reactions in terms of work-related burnout, client-related burnout, physical symptoms, and interpersonal strain at work. These findings emphasize the significant role of ambivalent identification, demonstrating how organizational (in)justice can lead to substantial impairments of psychological well-being. In line with previous findings, these results highlight the link between ambivalent identification and negative outcomes (Ciampa et al., 2019, 2021), supporting the notion that ambivalence represents a detrimental form of identification (Pratt, 2000). Overall, these results contribute to the research literature in two ways. First, although organizational justice was previously linked to employee stress and health, only a few studies explored the psychological mechanisms to explain this relationship (e.g., Judge and Colquitt, 2004), and none of them adopted a comprehensive theoretical framework that considers organizational contexts as social environments, emphasizing the context-dependent nature of groups and their function of social comparison (Turner et al., 1994; Hogg and Terry, 2000). The fact that ambivalent identification fully mediates the relationship between organizational justice and stress further confirms the importance of considering the role of social identities and their potential effects on employee health. Second, this represents the first empirical study considering organizational justice as a predictor of ambivalent identification. Although Ashforth et al. (2014) suggested numerous possibilities for future research questions about organizational triggers of ambivalence, proposing a comprehensive conceptual framework for their relationships, this is the first empirical attempt in that direction.

Some limitations should also be taken into account. First, despite the novelty of adopting a social identity perspective along with the ambivalence model in examining the link between organizational justice and stress, the data were gathered from a single source via self-report measures; this is a general potential limitation regarding the reliability of the measurements and their relationships. However, considering the nature of identification processes, it is recommended the use of self-report questionnaires for capturing individuals’ self-definition in terms of their unique traits and features (Ciampa et al., 2019). Therefore, future studies could include other sources of information regarding at least stress and health outcomes. A second limitation of the study is related to the cross-sectional nature of the data collection since this research design does not provide evidence of causality. This limitation could be addressed by replicating the present study using, ideally, longitudinal and experimental designs. However, the social identity model of stress (Haslam, 2004) and the multilevel perspective of ambivalence by Ashforth et al. (2014) are both in line with the causal direction suggested in the present study, which is the first that considers organizational justice as a trigger of ambivalent identification. A third limitation of the study relates to the generalizability of the findings. Because a convenience sampling method was used to collect the data, it was not possible to obtain more detailed information on the response rate or to control for variables that may have affected the response rate. Accordingly, although the participants were selected from among healthcare professionals, these findings cannot be strictly generalized to healthcare contexts without first extending the present results. Moreover, it is surprising that more than half of the participants in this study were doctors (55%), as the literature generally shows lower participation of doctors in research studies (see, e.g., Hummers-Pradier et al., 2008), mostly compared to nurses. Furthermore, given the size of the sample, these results indicate a trend that should be confirmed in future studies in the healthcare sector in order to generalize these findings, and these hypotheses could also be extended to other types of organizations.

Despite these limitations, this study contributes to future research questions: further research would benefit from the consideration of using different outcomes of stress and well-being, but also additional outcomes concerning cognitions and behaviors. Moreover, a different research design, such as a multilevel perspective, could explore the associations between ambivalence and organizational or group-level consequences, as proposed by Ashforth et al. (2014) model. Finally, future research should investigate organizational factors that determine, maintain, or facilitate ambivalent identification processes, broadening Kreiner and Ashforth’s (2004) empirical research which primarily focused on individual predictors, like intrarole conflict and breach of psychological contract. A fruitful investigation could also explore whether different facets of organizational justice, such as distributive, procedural, interpersonal, and informational justice (see, e.g., Greenberg and Colquitt, 2013) are differently related to ambivalent identification. The present study also offers practical implications. First, it can inform about the importance of reducing potential feelings of ambivalence toward the organization by implementing actions to minimize employees’ perceptions of unfair treatments within the organization. Organizations can allocate resources to develop strong positive identifications, adopting the social identity approach suggested by Haslam (2004). For example, improving a better communication climate (Smidts et al., 2001) and promoting equitable job conditions, like reducing short-term contracts (Johnson and Ashforth, 2008), or adopting the 5R program (Haslam et al., 2017) to foster stronger organizational and team identities, can help leaders and practitioners to reduce ambivalence toward the organization and its negative effects.

## Data availability statement

The raw data supporting the conclusions of this article will be made available by the author, without undue reservation.

## Ethics statement

All procedures performed in the study involving human participants were in accordance with the ethical standards of the institutional research committee (Department of Psychology, Sapienza University of Rome) and with the 1964 Helsinki Declaration and its later amendments or comparable ethical standards. The participants provided their written informed consent to participate in this study.

## Author contributions

VC: Conceptualization, Data curation, Formal analysis, Funding acquisition, Investigation, Methodology, Project administration, Resources, Supervision, Validation, Visualization, Writing – original draft, Writing – review & editing.
